# Mortality due to traumatic spinal cord injuries in Europe: a cross-sectional and pooled analysis of population-wide data from 22 countries

**DOI:** 10.1186/s13049-017-0410-0

**Published:** 2017-07-03

**Authors:** Marek Majdan, Dominika Plancikova, Eva Nemcovska, Lenka Krajcovicova, Alexandra Brazinova, Martin Rusnak

**Affiliations:** 1 Faculty of Health Sciences and Social Work, Department of Public Health, Trnava University, Univerzitne namestie 1, 91701 Trnava, Slovakia; 2 International Neurotrauma Research Organization, Moelkergasse 4, Vienna, Austria

**Keywords:** Traumatic spinal cord injury, Epidemiology, Mortality, Prevention, Death certificates, Cross-sectional analysis, Europe, Age-standardized mortality, Eurostat, Outcome

## Abstract

**Background:**

Traumatic spinal cord injuries (TSCI) pose a significant burden globally, while existing epidemiological data–especially on population mortality–are limited. The aim of this study was to calculate the age-standardized population mortality rates attributable to TSCI in 22 European countries, along with the pooled age-standardized mortality rate attributable to TSCI in Europe.

**Methods:**

A descriptive cross-sectional epidemiological study was conducted. Crude and age-standardized mortality rates attributable to TSCI for the year 2012 for 22 European countries were compared using data from death certificates provided by Eurostat. Pooled age-standardized mortality rates were calculated using the random effects model, and overall number of cases were estimated by extrapolating our findings to the populations of EU and Europe (48 countries), in 2012.

**Results:**

A total of 1840 TSCI-related deaths were identified, of which 1084 (59%) were males. The pooled age-standardized TSCI-related mortality rate of 6.7 per million (95% CI: 5.2 to 8.2) overall, 9.4 (95% CI: 7.3 to 11.5) for males, and 4.5 (95% CI: 3.4 to 5.6) for females. Extrapolating our results, 3152 (95% CI: 2441 to 3915) deaths would occur in 2012 in the EU-28 and 4570 (95% CI: 3538 to 5675) deaths in the whole Europe. TSCI-related deaths contributed by 2% (95% CI: 1.8% to 2.2%) to the overall injury related mortality. 61% of fatal TSCI were located in the cervical spine area.

**Conclusion:**

To our knowledge, this is the largest study that reports TSCI-related population-based mortalities to date which brings valuable information that can inform further research or prevention strategies. Our study presents a comprehensive and large-scale overview of TSCI-related population mortality in Europe. With an estimated toll of nearly five thousand lives that could be potentially saved by prevention, our findings confirm TSCI as an important cause of injury related deaths in Europe. Further action towards harmonization of case ascertainment and towards prevention strategies targeted mainly on the elderly is warranted.

**Electronic supplementary material:**

The online version of this article (doi:10.1186/s13049-017-0410-0) contains supplementary material, which is available to authorized users.

## Background

Traumatic spinal cord injuries (TSCI) remain a serious public health and social problem. While the incidence rates of TSCI are relatively low, it is a high-cost condition which has been associated with high mortality rates [1]. Along with skull-brain injury, TSCI resulted in the highest burden due to permanent disability in a study including six European countries [2]. They disrupt lives and come along with substantial human and societal toll [3]. Globally, they affect primarily the population of young males, and in high income countries – due to population aging – the elderly population [1, 3, 4]. Transport accidents (especially in low income countries with high increase of motor-vehicle use), falls (dominating in elderly populations of high income countries), and violence [1, 3] are globally among the three main causes of TSCI.

While world-wide epidemiological data are limited, it has been estimated that in 2007 between 133 and 226 thousands of incident cases of TSCI would occur resulting from accidents and violence (estimated global incidence rate of 23 per million) [1, 3]. Substantial variations have been observed between various regions of the world: a recent review reported an incidence of 40 per million inhabitants in US, 16 in Western Europe and 15 in Australia [3]. Another review reported incidence rates from single country studies ranging from 12.1 per million in The Netherlands to 57.8 per million in Portugal [5]. Incidence and mortality rates also differ between developing and developed countries [6]. The mortality risk in persons after TSCI largely depends on the level and severity of the injury in general. The victims are two to five times more likely to die prematurely than the general population [1]. A clear influence of age has been observed - much higher death rates were detected in elderly populations, compared to the young [4]. Similarly to incidence rates, substantial variation in population mortalities attributable to TSCI exist between countries or regions [1, 2, 6].

Improvements in prevention and outcome of TSCI must be supported by valid and timely epidemiological data. Several reviews have aimed to summarize the incidence and prevalence of TSCI, and their mortality globally [3–5, 7–9] – all reporting heterogeneity of findings across the studies. These could reflect true variations, but could also stem from varying methodological approaches, time-span, and case ascertainment used in the included studies. Such methodological differences are inherited in systematic reviews and may hinder the comparability of studies and the estimation of the burden of TSCI [7, 10]. In an endeavor to overcome these limitations, we present a cross-sectional analysis of population-based mortality rates, using data collected in a standardized manner and for the same time period.

The aim of this study was to calculate the age-standardized population mortality rates attributable to TSCI in 22 European countries, along with the pooled age-standardized mortality rate attributable to TSCI in Europe.

## Methods

### Study design, data sources and bias

A population-based cross-sectional epidemiological study was conducted using 2012 data from 22 European countries: Austria; Belgium; Croatia; Cyprus; Czech Republic; Denmark; Estonia; Finland; Germany; Ireland; Italy; Latvia; Lithuania; Netherlands; Portugal; Romania; Slovakia; Slovenia; Sweden; Switzerland; Turkey; and the United Kingdom. The inclusion of countries and the year of study was determined by data availability – the most recent year with the largest number of countries for which data were available was chosen.

All analyses are based on data obtained from Eurostat. The provided dataset contained a record for each death registered in the respective country, along with the cause of death that was derived from the death certificate. Eurostat routinely disseminates the aggregated data on causes of death classified using the European Shortlist for Causes of deaths which includes 86 causes [11, 12]. However, for the purpose of the study, both information on the external cause (E-code) and on the nature of injury (N-code) were provided for each death using the coding system of the 10th revision of the International Classification of Diseases and Related Health Problems (ICD-10) [13]. All data were provided as absolute numbers of deaths, broken down by sex and 5-year age groups.

Eurostat collects data on deaths from countries of the 28 member states of the EU, the former Yugoslav Republic of Macedonia, Albania, Iceland, Norway, Liechtenstein, and Switzerland under the framework of an EU regulation (No 328/2011) [14] which defines the scope, variables, and helps to ensure that the procedures of collecting and coding are harmonized and standardized to produce comparable results.

However, it must be noted that differences may exist in applying the recommended WHO’s updates in individual countries, or in the coverage of residents dying abroad or non-residents dying in the reporting country and these could - to a certain extent - affect the between-country comparability of the data. Furthermore, procedures on the level of responsible national authorities, or on the level of individuals may differ from country to country. All the above factors could result in selection bias. The authors of this study had no opportunity to control for such bias post-hoc.

### Case definition, variables and statistical methods

For the purposes of this study, TSCI-related deaths were defined by the set of ICD-10 codes exhibited in Table 1. In this definition, fractures of the spine were included, as it was assumed that if such fractures were fatal, they likely included a TSCI. Crude mortality rates were calculated using the mid-year population counts in the respective country and are given per million person years. Age-standardized mortality rates were calculated by the direct method of standardization using the European standard population [15] and are presented per million person years with 95% Confidence Intervals (95% CI).

**Table 1:** List of ICD-10 codes and their definitions used to define TSCI

Codes used to define fatalities due to TSCI	
ICD-10 Code	ICD-10 Definition
S14.0	Concussion and oedema of cervical spinal cord
S14.1	Other and unspecified injuries of cervical spinal cord
S14.2	Injury of nerve root of cervical spine
S24.0	Concussion and oedema of thoracic spinal cord
S24.1	Other and unspecified injuries of thoracic spinal cord
S24.2	Injury of nerve root of thoracic spine
S34.0	Concussion and oedema of lumbar spinal cord
S34.1	Other injury of lumbar spinal cord
S34.2	Injury of nerve root of lumbar and sacral spine
S12.0	Fracture of first cervical vertebra
S12.1	Fracture of second cervical vertebra
S12.2	Fracture of other specified cervical vertebra
S12.7	Multiple fractures of cervical spine
S22.0	Fracture of thoracic vertebra
S22.1	Multiple fractures of thoracic spine
S32.0	Fracture of lumbar vertebra
S32.1	Fracture of sacrum
S32.7	Multiple fractures of lumbar spine and pelvis
T91.3	Sequelae of injury of spinal cord

Rate ratios with 95% CI were used to quantify the relative differences in mortality rates between age groups and sexes. Proportions of TSCI-related deaths out of the overall number of injury-related deaths were calculated - for this purpose, deaths with unspecific causes (e.g. “Other and unspecified effects of external causes”), deaths caused by exposure to heat, frost, intoxications (T15-T65), and cases with other generalized causes (T66-T78; T80-T88) were excluded from the pool of injury related deaths.

Pooled mortality rates were estimated — to model possible heterogeneity of rates in the different countries, the random effects model was applied by the DerSimonian and Laird method. In order to assess the heterogeneity of pooled estimations, 95% prediction intervals and I^2^ were used. In order to allow for better comparability of our findings, pooled estimates were calculated based on crude rates and age-standardized rates. To estimate the total number of deaths attributable to TSCI in Europe, the crude pooled mortality rates were extrapolated to the population of the 28 member states of the EU and to the whole Europe (48 countries, as defined by the UN) [16].

The R statistical language and environment was used for all analyses in this study [17].

Additional data and results are presented in an online appendix – tables and figures in the appendix are denoted with A (e.g. Additional file 1: Table S1 and Additional file 1: Figure S1 etc.).

## Results

**Fig. 1:**
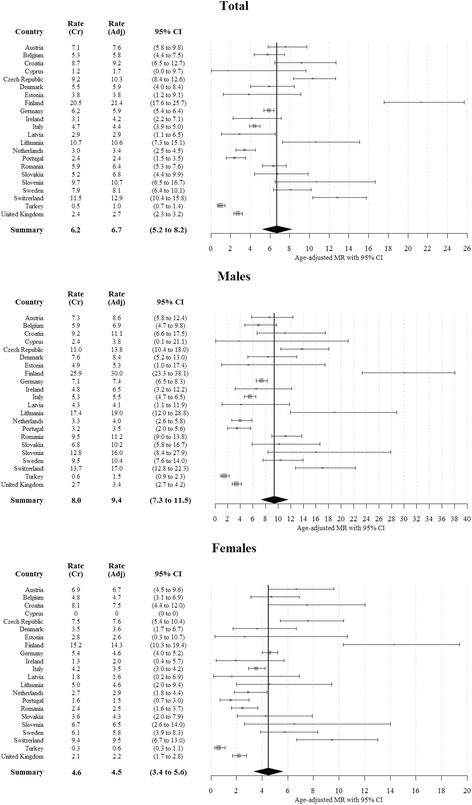
Crude and age-standardized TSCI-related mortality per million person years by country with pooled age-standardized estimate for 22 European countries. Males females combined: Prediction interval: 0 to 14.3; I^2^: 100%. Males: Prediction interval: 0 to 20.1; I^2^: 100%. Females: Prediction interval: 0 to 10.1; I^2^: 100%

A total of 1840 TSCI-related deaths were identified, of which 1084 (59%) were males. The age-standardized mortality rates ranged from 1.0 (95% CI: 0.7 to 1.4) in Turkey to 21.4 (95% CI: 17.6 to 25.7) in Finland. The mortality rates were in general higher in males, where they ranged from 1.5 (95% CI: 0.9 to 2.3) in Turkey to 30.0 (95% CI: 23.3 to 38.1) in Finland, compared to rates in females ranging from 0.6 (95% CI: 0.3 to 1.1) in Turkey to 14.3 (95% CI: 10.3 to 19.4) in Finland. Using the random effects model, pooled population mortality rates were estimated for Europe: based on age-standardized rates we estimated a mortality of 6.7 per million (95% CI: 5.2 to 8.2) overall, 9.4 (95% CI: 7.3 to 11.5) for males and 4.5 (95% CI: 3.4 to 5.6) for females; based on crude rates we estimated an overall pooled mortality rate of 6.2 (95% CI: 4.8 to 7.7), a pooled rate of 8.0 (95% CI: 6.1 to 9.8) in males, and 4.6 (95% CI: 3.6 to 5.6) in females. Substantial heterogeneity was observed between the countries in both sexes – I^2^ = 100%. See Fig. 1 for details and Additional file 1: Table S1 for an overview of crude and age-standardized TSCI-related population mortality rates by a country, sex and age group.

**Fig. 2:**
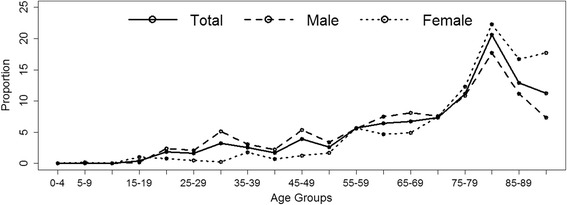
Proportions of TSCI-related deaths in age groups by sex, in percent of cases

Most deaths occurred in the older age groups, especially in the female population, where 83% of deaths were in the group of 65 years old or older; this proportion was 63% in males and 70% overall (ranging from 17% in Latvia to >90% in Switzerland, United Kingdom, or Cyprus). Figure 2 presents the proportions of deaths in age groups by sex and clearly demonstrates the shift towards older age groups, with a peak at 80–84 years of age in both sexes. In Table 2 and Additional file 1: Table S2 ratios of mortality rates are presented, quantifying the relative differences among age-groups (the age-group of 45–64 years old was chosen as the reference group and all ratios are in relation to the rate in this group). Clearly, mortality rates are substantially higher within the 65 years or older group, compared to all younger age groups. On average the rates in this group were higher by a factor of 9.5 when compared to the reference group, 1.7 in Romania, to 33.3 in Switzerland.

**Table 2:** Rate ratios of TSCI-related mortality rates per million person years by country and age groups with 95% CI, both sexes combined

	0–4	5–14	15–24	25–44	45–64	65+
Austria	-	-	0.3 (0.0–1.6)	-	Reference	8.5 (4.4–18.7)
Belgium	-	-	-	0.6 (0.2–2.0)	Reference	13.6 (5.9–39.9)
Croatia	-	-	-	0.5 (0.1–3.0)	Reference	11.8 (4.7–40.6)
Cyprus	-	-	-	-	-	8.9^a^
Czech Republic	-	-	0.7 (0.2–2.5)	1.0 (0.4–2.4)	Reference	11.7 (6.4–24.3)
Denmark	-	-	0.5 (0.0–3.0)	1.3 (0.4–4.5)	Reference	5.6 (2.3–17.3)
Estonia	-	-	-	1.8 (0.1–55.9)	Reference	2.8 (0.2–87.4)
Finland	-	-	0.4 (0.1–1.3)	0.7 (0.3–1.7)	Reference	9.3 (5.4–17.0)
Germany	0.2 (0.0–0.7)	-	0.4 (0.2–0.9)	0.5 (0.3–0.8)	Reference	12.6 (9.5–17.1)
Ireland	-	-	0.7 (0.0–6.0)	0.5 (0.1–3.3)	Reference	4.9 (1.4–23.8)
Italy	0.4 (0.0–1.8)	-	0.6 (0.2–1.7)	0.7 (0.3–1.5)	Reference	17.8 (11.4–29.9)
Latvia	-	-	-	0.3 (0.0–2.0)	Reference	0.4 (0.0–2.9)
Lithuania	-	-	1.0 (0.3–2.9)	0.6 (0.2–1.7)	Reference	1.7 (0.7–4.0)
Netherlands	-	-	-	-	Reference	10.2 (4.9–25.0)
Portugal	-	-	-	0.1 (0.0–0.6)	Reference	2.0 (0.9–4.6)
Romania	-	0.1 (0.0–0.3)	0.3 (0.1–0.7)	0.2 (0.1–0.4)	Reference	1.7 (1.1–2.5)
Slovakia	-	-	0.6 (0.1–2.6)	0.1 (0.0–0.8)	Reference	5.3 (2.3–13.8)
Slovenia	-	-	-	1.0 (0.1–9.6)	Reference	12.8 (3.6–87.8)
Sweden	-	-	0.2 (0.0–1.3)	0.6 (0.2–1.6)	Reference	8.8 (4.6–19.1)
Switzerland	-	-	-	0.5 (0.1–2.8)	Reference	33.3 (13.9–111.1)
Turkey	-	-	-	0.3 (0.1–1.0)	Reference	7.5 (3.5–18.0)
United Kingdom	-	-	-	0.2 (0.1–0.8)	Reference	17.1 (9.9–32.6)

^a^Crude rates, rate ratios could not be calculated

Table 3 presents the rate ratios of age-standardized mortality rates between sexes, and suggests that mortality rates in males are on average higher by a factor of 1.5 (95% CI: 1.4–1.6); rate ratios ranged from 1.1 in Austria and Croatia to 3.9 (95% CI: 2.5 to 6.2) in Romania. Thus, men were about 50% more likely to die of TSCI, compared to women in the analyzed countries.

**Table 3:** Rate ratios of TSCI-related mortality rates by sex with 95% CI

Country	Rate ratio (95% CI)
Austria	1..1 (0.6 to 1.8)
Belgium	1.2 (0.7 to 2.1)
Croatia	1.1 (0.6 to 2.2)
Cyprus	-
Czech Republic	1.5 (1.0 to 2.2)
Denmark	2.1 (1.0 to 4.7)
Estonia	1.7 (0.3 to 14.4)
Finland	1.7 (1.2 to 2.5)
Germany	1.3 (1.1 to 1.6)
Ireland	3.6 (1.1 to 16.7)
Italy	1.3 (1.0 to 1.6)
Latvia	2.3 (0.4 to 18.5)
Lithuania	3.5 (1.6 to 8.3)
Netherlands	1.2 (0.7 to 2.1)
Portugal	1.9 (0.9 to 4.6)
Romania	3.9 (2.5 to 6.2)
Slovakia	1.9 (0.9 to 4.3)
Slovenia	1.9 (0.8 to 5.1)
Sweden	1.6 (1.0 to 2.5)
Switzerland	1.5 (1.0 to 2.2)
Turkey	1.9 (1.0 to 3.9)
United Kingdom	1.3 (1.0 to 1.8)
TOTAL	1.5 (1.4 to 1.6)

Female mortality rates used as a reference category

**Fig. 3:**
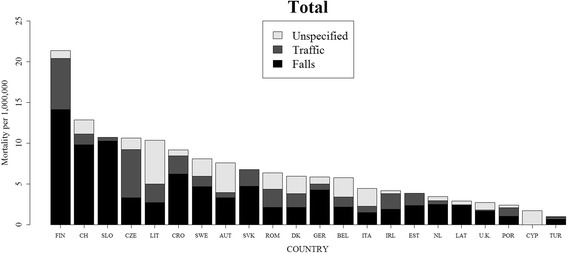
TSCI-related mortality rates by external cause in 22 European countries in 2012, both sexes combined. See Additional file 1: Figure S1 for data separately for males and females

The most common recorded external causes of injury were falls and traffic accidents. On average, 53% of deaths occurred after a fall and 23% after a traffic accident. These proportions were similar in both sexes: on average 51% of deaths occurred after falls in males and 58% in females; 25% of deaths were traffic related in males and 20% in females. Figure 3 presents a graphical overview of age-standardized mortality rates by external cause for each country and confirms the dominance of falls as the primary external cause (see Additional file 1: Figure S1 for sex-specific data).

**Fig. 4:**
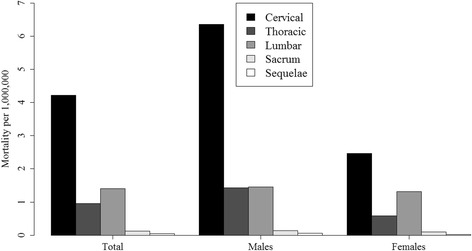
TSCI-related mortality rates by level of injury, in 22 European countries in 2012, both sexes combined. See Additional file 1: Figure S2 for data separately for males and females

The most common site of fatal TSCI overall was the cervical area where 61% of all cases were located (66% in males and 55% in females), followed by lumbar TSCI with 23% (19% males, 26% females) and thoracic TSCI contributing 14% overall, 14% in males and 16% in females. Expressed in rates, cervical TSCI was associated with an overall mortality of 4.22, lumbar TSCI 1.4 and thoracic TSCI with a mortality of 0.95 per million. See details in Fig. 4 and in Additional file 1: Figure S2 where the site distributions are presented for each country separately.

To evaluate the potential influence of the general economy level of the country on the observed heterogeneity between mortality rates, we have categorized the included countries based on their GDP per capita to a group with a per-capita GDP of <45,000 USD and a group with per-capita GDP of ≥45,000 USD; we found a pooled crude rate of 5.3 (95% CI: 3.8 to 6.3) per million in countries with lower GDP, and a pooled rate of 8.4 (95% CI: 5.0 to 11.8) in countries with higher economy level.

**Fig. 5:**
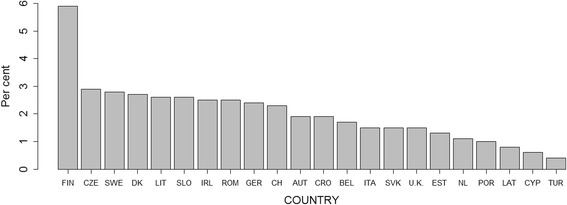
Proportions of age-standardized TSCI related mortality rates out of all-injury mortality rates in 22 European countries in 2012, both sexes combined

TSCI-related deaths were a relatively minor contributor to the overall injury-related mortality (Table 4 and Fig. 5) with proportions ranging from <1% in Cyprus, Latvia or Turkey up to 5.9% (95% CI: 5.0% to 6.8%) in Finland – on average they contributed 2% (95% CI: 1.8% to 2.2%). When extrapolating the pooled crude TSCI-mortality rates from the analyzed 22 countries to the whole population of the EU-28, in 2012 a total of 3152 (95% CI: 2441 to 3915) TSCI deaths would occur. The number of deaths in the population of the whole Europe (48 countries) would be 4570 (95% CI: 3538 to 5675). See Table 5 and Additional file 1: Table S3 for details.

**Table 4:** Number of deaths^a^ and age-standardized mortality rates per million person years due to all injuries and TSCI with proportions of TSCI-related age-standardized mortalities, stratified by country

Country	TSCI		ALL INJURIES		Percent TSCI MR/All injury MR^b^ (95% CI)
	Number of deaths	Age-standardized rate (95% CI)	Number of deaths	Age-standardized rate (95% CI)	
Austria	60	7.6 (5.8 to 9.8)	3181	392.2 (378.6 to 406.1)	1.9 (1.5 to 2.4)
Belgium	59	5.8 (4.4 to 7.5)	3558	340.5 (329.4 to 351.9)	1.7 (1.3 to 2.1)
Croatia	37	9.2 (6.5 to 12.7)	2033	493.6 (472.3 to 515.6)	1.9 (1.4 to 2.5)
Cyprus	1	1.7 (0.0 to 9.7)	222	309.6 (269.0 to 355.0)	0.6 (0.0 to 2.7)
Czech Republic	97	10.3 (8.4 to 12.6)	3538	360.0 (348.1 to 372.1)	2.9 (2.4 to 3.4)
Denmark	31	5.9 (4.0 to 8.4)	1143	218.7 (206.2 to 231.8)	2.7 (2.0 to 3.6)
Estonia	5	3.8 (1.2 to 9.1)	384	293.5 (264.9 to 324.5)	1.3 (0.5 to 2.8)
Finland	111	21.4 (17.6 to 25.7)	1919	364.1 (348.0 to 380.8)	5.9 (5.0 to 6.8)
Germany	509	5.9 (5.4 to 6.4)	21,229	246.4 (243.1 to 249.8)	2.4 (2.2 to 2.6)
Ireland	14	4.2 (2.2 to 7.1)	592	169.3 (155.6 to 184.0)	2.5 (1.4 to 3.9)
Italy	282	4.4 (3.9 to 5.0)	18,922	301.0 (296.7 to 305.3)	1.5 (1.3 to 1.6)
Latvia	6	2.9 (1.1 to 6.5)	755	367.7 (341.9 to 395.1)	0.8 (0.3 to 1.7)
Lithuania	32	10.6 (7.3 to 15.1)	1242	417.4 (394.4 to 441.3)	2.6 (1.8 to 3.4)
Netherlands	50	3.4 (2.5 to 4.5)	4612	308.7 (299.9 to 317.8)	1.1 (0.8 to 1.4)
Portugal	25	2.4 (1.5 to 3.5)	2635	249.5 (240.0 to 259.2)	1.0 (0.6 to 1.4)
Romania	118	6.4 (5.3 to 7.6)	4987	258.4 (251.2 to 265.7)	2.5 (2.1 to 2.9)
Slovakia	28	6.8 (4.4 to 9.9)	2072	454.0 (434.1 to 474.6)	1.5 (1.0 to 2.1)
Slovenia	20	10.7 (6.5 to 16.7)	793	417.5 (388.9 to 447.9)	2.6 (1.7 to 3.7)
Sweden	75	8.1 (6.4 to 10.1)	2658	285.7 (275.0 to 296.8)	2.8 (2.3 to 3.4)
Switzerland	92	12.9 (10.4 to 15.8)	4058	549.5 (532.7 to 566.8)	2.3 (1.9 to 2.8)
Turkey	35	1.0 (0.7 to 1.4)	11,096	220.5 (215.8 to 225.2)	0.4 (0.3 to 0.6)
United Kingdom	153	2.7 (2.3 to 3.2)	10,653	183.2 (179.8 to 186.8)	1.5 (1.3 to 1.7)
Overall^c^	1840	6.7 (5.2 to 8.2)	102,282	327.3 (285.6 to 369.0)	2.0 (1.8 to 2.2)

*TSCI* Traumatic Spinal Cord Injury, *CI* Confidence Interval

^a^We only included those cases where the cause of death has been defined by a specific ICD diagnostic code; cases where the cause was defined as “other and unspecified effects of external causes” or any of the ICD codes of T15-T78 and T80-T88 were excluded

^b^Percent of age-standardized TSCI related mortality rate out of age-standardized all injury related mortality rate

^c^Pooled age-standardized mortality rates estimated using the random effects model

**Table 5:** Estimated numbers of TSCI-related deaths in the EU-28 and in the whole Europe based on extrapolation of pooled crude mortality rates

	Population Count	Pooled crude mortality rate with 95% CI		Estimated number of cases with 95% CI	
Deaths due to TSCI					
EU-28^27^	508,450,856	6.2	(4.8 to 7.7)	3152	(2441 to 3915)
Europe^30a^	737,021,812			4570	(3538 to 5675)

*CI* Confidence Interval

^a^Europe as defined by the UN population division including 48 countries

## Discussion

### Main findings

We conducted a population-based cross-sectional study using data obtained from Eurostat in order to calculate population-based mortality rates attributable to TSCI in 22 European countries in 2012. We identified a total of 1840 TSCI-related deaths were identified, of which 1084 (59%) were males. We found a pooled age-standardized TSCI-related mortality rate of 6.7 per million (95% CI: 5.2 to 8.2) overall, 9.4 (95% CI: 7.3 to 11.5) for males, and 4.5 (95% CI: 3.4 to 5.6) for females. Extrapolating these results, 3152 (95% CI: 2441 to 3915) deaths would occur in 2012 in the EU-28 and 4570 (95% CI: 3538 to 5675) deaths in the whole Europe. TSCI-related deaths contributed by 2% (95% CI: 1.8% to 2.2%) to the overall injury related mortality. 61% of fatal TSCI were located in the cervical spine area. To our knowledge, this is the largest study that reports TSCI-related population-based mortalities to date which brings valuable information that can inform further research or prevention strategies.

### Comparison to other studies and interpretation

Despite the relatively large number of published studies dealing with epidemiology of TSCI, studies reporting population-based mortality rates are rare. A study from the US state of South Carolina reported a crude mortality rate of 27.4 per million between 1981 and 1998 [18], whereas a study from Austria reported an average crude mortality rate of 4.1 and an age-standardized mortality rate of 2.38 (95% CI: 1.61 to 6.63) per million for the period of 2002–2012 [19]. The relatively large difference between the results in these two studies may stem from the different study periods, but could also suggest varying methodological approaches – our pooled estimates fall in-between them. Most of the other published studies focus on incidence or prevalence, and report mortality rates or deaths as standardized mortality ratios [5, 20–23], percent out of total numbers of patients admitted to a hospital after TSCI [21, 24], or life expectancy and survival of patients years after injury [4, 5, 8, 23, 25–27], and thus they are not directly comparable to these results.

One of the key findings of this paper is a relatively large variation of mortality rates that is observed, despite analyzing data for the same time period which were collected under unified standards for definitions and procedures. These could reflect true differences between the analyzed countries due to various levels of implementation of preventive measures, varying level of health care, or in general a varying level of risk of TSCI between the populations of these countries. However, there are other factors which might be driving these differences and they must be considered when interpreting these findings.

First, there might be various coding practices within countries or even within institutions, which could influence the overall picture of causes of deaths in the respective country and in Europe. An example of the influence of such practices might be the differing proportions of deaths coded as multiple injuries. Countries reporting large proportions of deaths caused by multiple injury tend to have lower TSCI-mortality rates, indicating that some deaths that were actually caused by TSCI could be ‘hidden’ under these categories. For example, the lowest TSCI related mortality in our study was observed in Turkey which reported 49% of injury related deaths being due to a multiple injury, whereas Finland with the highest TSCI mortality attributed only 5% of deaths to multiple injuries (on average, the 22 countries reported 22% of injury related deaths as caused by multiple injury).

Second, it has been previously reported that nations with similar economies tend to have similar patterns of TSCI epidemiology [7]. Such a difference was found also in this study (pooled crude rate of 5.3 per million in countries with lower GDP, and 8.4 in countries with higher economy level). Although more detailed investigation is needed to provide stronger evidence, it is suggested that some of the between country variability of mortality rates could be explained by varying GDP of the respective countries.

The observed between-country heterogeneity of our findings is in line with the heterogeneity reported for incidence or prevalence rates globally [3–5, 7, 9]. These findings together could suggest that both the risk of attaining a TSCI, and dying from it differs quite substantially between the countries; this could be a result of demographic differences, but could also reflect the level of attention that is devoted to prevention in the respective country.

### Bias and limitations

There are limitations to this study that we acknowledge. Different epidemiological studies use different definitions for case ascertainment. Therefore, a potential limitation of this study is that the sensitivity of the ICD-10 codes used to identify cases and deaths was not analyzed, nor compared to other definitions. However, it has been shown that using ICD-10 coding to define TSCI is superior to previous versions of the ICD [28].

This study analyzed data from 22 countries and it is possible that differences in practices of coding the causes of death between them introduced a certain level of selection bias. We are aware of this limitation, but were not able to control for it, as we had no access to the death certificates themselves. However, the data were submitted by each country to Eurostat under specific guidelines included in an EU-regulation and we assume that this setup helped achieving the highest possible degree of harmonization that was possible under the circumstances.

In addition, we were not able to calculate incidence rates for the included countries, as data that would allow this were not available. As a consequence, we were not able to put the reported mortality rates in context with the overall occurrence of TSCI in the respective countries. Doing so would help interpreting our results and the heterogeneity that was observed between the mortality rates in our study.

Certain aspects of our findings, such as the age distribution of deaths may be to a certain extent biased by the varying period between the injury itself and the time of death. Our results should be interpreted with this in mind.

There is substantial heterogeneity in our pooled estimates (as documented by high I^2^) which should be taken into account when interpreting them.

## Conclusions

Our study presents a comprehensive and large-scale overview of TSCI-related population mortality in Europe. With an estimated toll of nearly five thousand lives that could be potentially saved by prevention, our findings confirm TSCI as an important cause of injury related deaths in Europe. Further action towards harmonization of case ascertainment and towards prevention strategies targeted mainly on the elderly is warranted.

## Additional file

Additional file 1: Table S1. Crude and age-standardized TSCI-related mortality rates per million person years by country and age groups (total). **Table S2**. Rate ratios of TSCI-related mortality rates per million person years by country and age groups with 95% CI by sex. **Table S3**. Number of deaths* and age-standardized mortality rates per million person years due to all injuries and TSCI with proportions of TSCI-related age-standardized mortalities and number of deaths, stratified by country and sex. **Figure S1**. TSCI-related mortality rates by external cause in 22 European countries in 2012, by sex. **Figure S2**. TSCI-related mortality rates by level of injury, in 22 European countries in 2012, by sex. (PDF 5 kb)

## Abbreviations

CI: Confidence Interval
GDP: Gross Domestic Product
ICD: International Classification of Diseases
TSCI: Traumatic Spinal Cord Injury
