# Supplementary material for: Mortality due to traumatic spinal cord injuries in Europe: a cross-sectional and pooled analysis of population-wide data from 22 countries
Source: Scand J Trauma Resusc Emerg Med. 2017 Jul 3;25:64. doi: 10.1186/s13049-017-0410-0 (PMC5496373; doi:10.1186/s13049-017-0410-0)
Supplement: Additional file 1: Table S1. — Crude and age-standardized TSCI-related mortality rates per million person years by country and age groups (total). Table S2. Rate ratios of TSCI-related mortality rates per million person years by country and age groups with 95% CI by sex. Table S3. Number of deaths* and age-standardized mortality rates per million person years due to all injuries and TSCI with proportions of TSCI-related age-standardized mortalities and number of deaths, stratified by country and sex. Figure S1. TSCI-related mortality rates by external cause in 22 European countries in 2012, by sex. Figure S2. TSCI-related mortality rates by level of injury, in 22 European countries in 2012, by sex. (PDF 5 kb) [file 13049_2017_410_MOESM1_ESM.pdf]

Traumatic spinal cord injury-related deaths in Europe: a cross-sectional and pooled analysis of population-wide data from 22 countries

Authors:

Marek Majdan[1,2], Dominika Plancikova[1], Eva Nemcovska[1], Lenka Krajcovicova[1], Alexandra Brazinova[1,2], Martin Rusnak[1]

Affiliations:

1: Trnava University, Faculty of Health Sciences and Social Work, Department of Public Health, Univerzitne namestie 1, 91701 Trnava, Slovakia, Tel: +421-33-5939495 (206), Fax: +421-33-5939555

2: International Neurotrauma Research Organization, Moelkergasse 4, Vienna, Austria

Corresponding Author

Dr. Marek Majdan, PhD

Trnava University

Faculty of Health Sciences and Social Work

Department of Public Health

Univerzitne namestie 1

91701 Trnava

Slovakia

Tel: +421-33-5939495 (206)

Fax: +421-33-5939555

mmajdan@truni.sk, majdan@email.com

Table S1. Crude and age-standardized TSCI-related mortality rates per million person years by country and age groups (total)

Sex	Country	0 to 4	5 to 14	15 to 24	25 to 44	45 to 64	65 +	Crude Rate	Age-Standardized Rate (95% CI)
Total	**Austria**	0	0	1.0	0	3.8	33.1	7.1	7.6 (5.8 to 9.8)
	**Belgium**	0	0	0	1.7	2.7	23.7	5.3	5.8 (4.4 to 7.5)
	**Croatia**	0	0	0	1.7	3.3	40.3	8.7	9.2 (6.5 to 12.7)
	**Cyprus**	0	0	0	0	0	8.9	1.2	1.7 (0.0 to 9.7)
	**Czech Republic**	0	0	2.5	3.4	3.5	42.1	9.2	10.3 (8.4 to 12.6)
	**Denmark**	0	0	1.4	4.2	3.3	19.3	5.5	5.9 (4.0 to 8.4)
	**Estonia**	0	0	0	5.4	2.9	8.5	3.8	3.8 (1.2 to 9.1)
	**Finland**	0	0	3.0	6.7	9.2	86.1	20.5	21.4 (17.6 to 25.7)
	**Germany**	0.3	0	0.9	0.9	2.0	25.5	6.2	5.9 (5.4 to 6.4)
	**Ireland**	0	0	1.8	1.4	2.8	14.5	3.1	4.2 (2.2 to 7.1)
	**Italy**	0.4	0	0.7	0.8	1.1	19.7	4.7	4.4 (3.9 to 5.0)
	**Latvia**	0	0	0	1.8	7.2	2.6	2.9	2.9 (1.1 to 6.5)
	**Lithuania**	0	0	12.2	7.7	12.2	20.3	10.7	10.6 (7.3 to 15.1)
	**Netherlands**	0	0	0	0	1.5	15.5	3.0	3.4 (2.5 to 4.5)
	**Portugal**	0	0	0	0.3	3.5	6.9	2.4	2.4 (1.5 to 3.5)
	**Romania**	0	0.5	2.9	2.1	9.1	15.4	5.9	6.4 (5.3 to 7.6)
	**Slovakia**	0	0	2.8	0.6	4.8	25.7	5.2	6.8 (4.4 to 9.9)
	**Slovenia**	0	0	0	3.3	3.4	45.9	9.7	10.7 (6.5 to 16.7)
	**Sweden**	0	0	0.8	2.0	3.7	33.2	7.9	8.1 (6.4 to 10.1)
	**Switzerland**	0	0	0	0.9	1.8	62.2	11.5	12.9 (10.4 to 15.8)
	**Turkey**	0	0	0	0.2	0.5	4.1	0.5	1.0 (0.7 to 1.4)
	**United Kingdom**	0	0	0	0.2	0.7	12.8	2.4	2.7 (2.3 to 3.2)
Males	**Austria**	0	0	1.9	0	5.1	36.2	7.3	8.6 (5.8 to 12.4)
	**Belgium**	0	0	0	3.3	4.0	25.3	5.9	6.9 (4.7 to 9.8)
	**Croatia**	0	0	0	3.4	6.7	43.1	9.2	11.1 (6.6 to 17.5)
	**Cyprus**	0	0	0	0	0	19.4	2.4	3.8 (0.1 to 21.1)
	**Czech Republic**	0	0	1.6	5.5	6.4	53.7	11.0	13.8 (10.4 to 18.0)
	**Denmark**	0	0	2.7	7.0	5.3	24.8	7.6	8.4 (5.2 to 13.0)
	**Estonia**	0	0	0	10.6	0	12.7	4.9	5.3 (1.0 to 17.4)
	**Finland**	0	0	3.0	11.6	13.2	118.6	25.9	30.0 (23.3 to 38.1)
	**Germany**	0	0	1.5	1.6	3.4	30.2	7.1	7.4 (6.5 to 8.3)
	**Ireland**	0	0	3.6	2.8	5.7	19.7	4.8	6.5 (3.2 to 12.2)
	**Italy**	0.7	0	1.0	1.2	1.9	23.3	5.3	5.5 (4.7 to 6.5)
	**Latvia**	0	0	0	3.6	12.0	0	4.3	4.1 (1.1 to 11.9)
	**Lithuania**	0	0	14.3	13.0	24.2	38.3	17.4	19.0 (12.0 to 28.8)
	**Netherlands**	0	0	0	0	2.5	17.0	3.3	4.0 (2.6 to 5.8)
	**Portugal**	0	0	0	0.7	4.4	10.8	3.2	3.5 (2.0 to 5.6)
	**Romania**	0	0	4.8	3.2	16.0	28.3	9.5	11.2 (9.0 to 13.8)
	**Slovakia**	0	0	5.5	1.2	7.0	37.8	6.8	10.2 (5.8 to 16.7)
	**Slovenia**	0	0	0	6.4	6.6	64.5	12.8	16.0 (8.4 to 27.9)
	**Sweden**	0	0	1.6	1.6	6.6	41.6	9.5	10.4 (7.6 to 14.0)
	**Switzerland**	0	0	0	1.8	3.6	80.1	13.7	17.0 (12.8 to 22.3)
	**Turkey**	0	0	0	0.2	0.8	6.2	0.6	1.5 (0.9 to 2.3)
	**United Kingdom**	0	0	0	0.4	1.1	15.3	2.7	3.4 (2.7 to 4.2)
Females	**Austria**	0	0	0	0	2.5	30.8	6.9	6.7 (4.5 to 9.6)
	**Belgium**	0	0	0	0	1.3	22.5	4.8	4.7 (3.1 to 6.9)
	**Croatia**	0	0	0	0	0	38.5	8.1	7.5 (4.4 to 12.0)
	**Cyprus**	0	0	0	0	0	0	0	0
	**Czech Republic**	0	0	3.4	1.3	0.7	34.1	7.5	7.6 (5.4 to 10.4)
	**Denmark**	0	0	0	1.4	1.3	14.8	3.5	3.6 (1.7 to 6.7)
	**Estonia**	0	0	0	0	5.3	6.3	2.8	2.6 (0.3 to 10.7)
	**Finland**	0	0	3.1	1.5	5.2	62.4	15.2	14.3 (10.3 to 19.4)
	**Germany**	0.6	0	0.2	0.2	0.7	22.0	5.4	4.6 (4.0 to 5.2)
	**Ireland**	0	0	0	0	0	10.0	1.3	2.0 (0.4 to 5.7)
	**Italy**	0	0	0.3	0.4	0.4	17.0	4.2	3.5 (3.0 to 4.2)
	**Latvia**	0	0	0	0	3.3	3.9	1.8	1.6 (0.2 to 6.9)
	**Lithuania**	0	0	10.0	2.5	2.3	11.1	5.0	4.6 (2.0 to 9.4)
	**Netherlands**	0	0	0	0	0.4	14.3	2.7	2.9 (1.8 to 4.4)
	**Portugal**	0	0	0	0	2.7	4.2	1.6	1.5 (0.7 to 3.0)
	**Romania**	0	1.0	0.9	1.0	2.6	6.7	2.4	2.5 (1.6 to 3.7)
	**Slovakia**	0	0	0	0	2.7	18.4	3.6	4.3 (2.0 to 7.9)
	**Slovenia**	0	0	0	0	0	33.5	6.7	6.5 (2.6 to 14.0)
	**Sweden**	0	0	0	1.7	0.8	26.3	6.1	5.8 (3.9 to 8.3)
	**Switzerland**	0	0	0	0	0	48.5	9.4	9.5 (6.7 to 13.0)
	**Turkey**	0	0	0	0.2	0.3	2.5	0.3	0.6 (0.3 to 1.1)
	**United Kingdom**	0	0	0	0	0.4	10.7	2.1	2.2 (1.7 to 2.8)

CI: Confidence Interval;

Table S2. Rate ratios of TSCI-related mortality rates per million person years by country and age groups with 95% CI by sex

Sex	Country	0 - 4	5 - 14	15 - 24	25 - 44	45 - 64	65+
Males	**Austria**	-	-	0.4 (0.0 - 2.5)	-	Reference	6.9 (3.0 - 18.9)
	**Belgium**	-	-	-	0.8 (0.2 - 2.9)	Reference	6.2 (2.6 - 17.1)
	**Croatia**	-	-	-	0.5 (0.1 - 2.9)	Reference	6.2 (2.2 - 22.7)
	**Cyprus**	-	-	-	-	-	19.4*
	**Czech Republic**	-	-	0.3 (0.0 - 1.6)	0.8 (0.3 - 2.2)	Reference	8.2 (4.2 - 18.3)
	**Denmark**	-	-	0.6 (0.0 - 4.1)	1.3 (0.3 - 5.5)	Reference	4.5 (1.5 - 16.9)
	**Estonia**	-	-	-	Reference	-	1.3 (0.0 - 15.7)
	**Finland**	-	-	0.3 (0.0 - 1.3)	0.9 (0.3 - 2.3)	Reference	8.9 (4.7 - 18.6)
	**Germany**	-	-	0.5 (0.2 - 1.0)	0.5 (0.3 - 0.8)	Reference	8.9 (6.5 - 12.6)
	**Ireland**	-	-	0.7 (0.0 - 5.9)	0.5 (0.1 - 3.4)	Reference	3.4 (0.8 - 17.6)
	**Italy**	0.4 (0.0 - 2.1)	-	0.6 (0.1 - 1.7)	0.7 (0.3 - 1.5)	Reference	12.4 (7.5 - 22.1)
	**Latvia**	-	-	-	0.3 (0.0 - 2.8)	Reference	-
	**Lithuania**	-	-	0.6 (0.1 - 2.1)	0.5 (0.2 - 1.6)	Reference	1.6 (0.6 - 4.3)
	**Netherlands**	-	-	-	-	Reference	6.6 (2.8 - 18.1)
	**Portugal**	-	-	-	0.2 (0.0 - 1.1)	Reference	2.4 (0.9 - 7.3)
	**Romania**	-	-	0.3 (0.1 - 0.7)	0.2 (0.1 - 0.4)	Reference	1.8 (1.1 - 2.8)
	**Slovakia**	-	-	0.8 (0.1 - 3.9)	0.2 (0.0 - 1.2)	Reference	5.3 (1.8 - 17.4)
	**Slovenia**	-	-	-	1.0 (0.1 - 9.3)	Reference	9.2 (2.3 - 65.9)
	**Sweden**	-	-	0.3 (0.0 - 1.5)	0.3 (0.0 - 1.1)	Reference	6.2 (3.0 - 14.6)
	**Switzerland**	-	-	-	0.5 (0.1 - 2.8)	Reference	21.6 (8.8 - 72.9)
	**Turkey**	-	-	-	0.2 (0.0 - 1.0)	Reference	7.4 (3.0 - 21.1)
	**United Kingdom**	-	-	-	0.3 (0.1 - 1.1)	Reference	13.4 (7.1 - 29.0)
Females	**Austria**	-	-	-	-	Reference	11.7 (4.1 - 50.7)
	**Belgium**	-	-	-	-	Reference	15.7 (4.7 - 105.2)
	**Croatia**	-	-	-	-	-	38.5*
	**Cyprus**	-	-	-	-	-	-
	**Czech Republic**	-	-	4.6 (0.4 - 144.9)	1.7 (0.1 - 54.1)	Reference	42.7 (9.4 - 999.7)
	**Denmark**	-	-	-	1.1 (0.0 - 41.3)	Reference	9.8 (1.8 - 245.8)
	**Estonia**	-	-	-	-	Reference	1.2 (0.0 - 46.6)
	**Finland**	-	-	0.7 (0.0 - 4.7)	0.3 (0.0 - 2.3)	Reference	11.5 (4.6 - 39.3)
	**Germany**	1.0 (0.0 - 5.7)	-	0.4 (0.0 - 2.1)	0.3 (0.0 - 1.3)	Reference	32.5 (17.2 - 72.2)
	**Ireland**	-	-	-	-	-	10.0*
	**Italy**	-	-	1.1 (0.0 - 9.1)	1.0 (0.2 - 6.0)	Reference	45.3 (17.2 - 190.2)
	**Latvia**	-	-	-	-	Reference	1.2 (0.0 - 45.9)
	**Lithuania**	-	-	4.2 (0.3 - 131.2)	1.1 (0.0 - 44.0)	Reference	4.5 (0.6 - 122.0)
	**Netherlands**	-	-	-	-	Reference	29.4 (6.2 - 697.6)
	**Portugal**	-	-	-	-	Reference	1.6 (0.4 - 6.5)
	**Romania**	-	0.4 (0.0 - 2.4)	0.4 (0.0 - 2.1)	0.4 (0.1 - 1.5)	Reference	2.5 (1.0 - 6.8)
	**Slovakia**	-	-	-	-	Reference	6.5 (1.6 - 47.6)
	**Slovenia**	-	-	-	-	-	33.5*
	**Sweden**	-	-	-	1.9 (0.2 - 58.9)	Reference	27.7 (6.0 - 655.1)
	**Switzerland**	-	-	-	-	-	48.5*
	**Turkey**	-	-	-	0.6 (0.1 - 6.2)	Reference	8.9 (2.2 - 64.8)
	**United Kingdom**	-	-	-	-	Reference	27.9 (10.4 - 117.8)

** Crude rates, rate ratios could not be calculated*

Table S3. Number of deaths* and age-standardized mortality rates per million person years due to all injuries and TSCI with proportions of TSCI-related age-standardized mortalities and number of deaths, stratified by country and sex

Sex	Country	TSCI		ALL INJURIES		Percent TSCI MR/ All injury MR** (95% CI)
		Number of deaths	Age-standardized rate (95% CI)	Number of deaths	Age-standardized rate (95% CI)	
Males	Austria	30	8.6 (5.8 to 12.4)	1946	522.1 (498.9 to 546.1)	1.7 (1.2 to 2.3)
	Belgium	32	6.9 (4.7 to 9.8)	2056	419.6 (401.4 to 438.3)	1.6 (1.2 to 2.2)
	Croatia	19	11.1 (6.6 to 17.5)	1190	644.9 (608.2 to 683.4)	1.7 (1.1 to 2.6)
	Cyprus	1	3.8 (0.1 to 21.1)	143	392.4 (328.0 to 466.6)	1.0 (0.0 to 4.5)
	Czech Republic	57	13.8 (10.4 to 18.0)	2318	504.0 (438.2 to 525.6)	2.7 (2.2 to 3.4)
	Denmark	21	8.4 (5.2 to 13.0)	612	245.1 (226.0 to 265.5)	3.4 (2.3 to 4.9)
	Estonia	3	5.3 (1.0 to 17.4)	284	491.4 (434.5 to 554.4)	1.1 (0.2 to 3.1)
	Finland	69	30.0 (23.3 to 38.1)	1194	492.1 (464.3 to 521.1)	6.1 (5.0 to 7.3)
	Germany	285	7.4 (6.5 to 8.3)	12009	302.1 (296.7 to 307.6)	2.4 (2.2 to 2.7)
	Ireland	11	6.5 (3.2 to 12.2)	372	212.8 (190.8 to 236.7)	3.1 (1.7 to 5.1)
	Italy	153	5.5 (4.7 to 6.5)	10277	363.8 (356.8 to 370.9)	1.5 (1.3 to 1.7)
	Latvia	4	4.1 (1.1 to 11.9)	548	608.3 (557.4 to 663.0)	0.7 (0.2 to 1.8)
	Lithuania	24	19.0 (12.0 to 28.8)	898	697.7 (651.8 to 746.3)	2.7 (1.8 to 3.9)
	Netherlands	27	4.0 (2.6 to 5.8)	2279	319.8 (306.7 to 333.4)	1.2 (0.9 to 1.8)
	Portugal	16	3.5 (2.0 to 5.6)	1818	379.6 (362.3 to 397.6)	0.9 (0.5 to 1.4)
	Romania	93	11.2 (9.0 to 13.8)	3824	424.6 (410.9 to 438.6)	2.6 (2.2 to 3.1)
	Slovakia	18	10.2 (5.8 to 16.7)	1371	645.6 (609.4 to 683.6)	1.6 (1.0 to 2.4)
	Slovenia	13	16.0 (8.4 to 27.9)	424	483.7 (437.7 to 533.5)	3.3 (1.9 to 5.2)
	Sweden	45	10.4 (7.6 to 14.0)	1521	347.6 (330.3 to 365.6)	3.0 (2.3 to 3.8)
	Switzerland	54	17.0 (12.8 to 22.3)	2218	644.2 (617.4 to 672.0)	2.6 (2.1 to 3.3)
	Turkey	23	1.5 (0.9 to 2.3)	7926	306.3 (298.4 to 314.4)	0.5 (0.3 to 0.7)
	United Kingdom	86	3.4 (2.7 to 4.2)	5929	214.4 (208.9 to 219.9)	1.6 (1.3 to 1.9)
	**Overall*** **	**1084**	**9.4 (7.3 to 11.5)**	**61157**	**439.2 (375.5 to 502.9)**	**2.1 (1.9 to 2.3)**
Females	Austria	30	6.7 (4.5 to 9.6)	1235	275.9 (260.7 to 291.8)	2.4 (1.7 to 3.3)
	Belgium	27	4.7 (3.1 to 6.9)	1502	263.7 (250.5 to 277.4)	1.8 (1.2 to 2.5)
	Croatia	18	7.5 (4.4 to 12.0)	843	354.9 (331.3 to 379.8)	2.1 (1.3 to 3.2)
	Cyprus	0	0	79	231.6 (182.6 to 289.8)	0
	Czech Republic	40	7.6 (5.4 to 10.4)	1220	230.0 (217.2 to 243.3)	3.3 (2.5 to 4.3)
	Denmark	10	3.6 (1.7 to 6.7)	531	191.0 (175.1 to 208.0)	1.9 (1.0 to 3.2)
	Estonia	2	2.6 (0.3 to 10.7)	100	133.6 (108.6 to 162.9)	2.0 (0.3 to 6.6)
	Finland	42	14.3 (10.3 to 19.4)	725	248.6 (230.8 to 267.4)	5.8 (4.5 to 7.3)
	Germany	224	4.6 (4.0 to 5.2)	9220	192.5 (188.5 to 196.4)	2.4 (2.1 to 2.7)
	Ireland	3	2.0 (0.4 to 5.7)	220	127.7 (111.1 to 146.1)	1.5 (0.4 to 3.9)
	Italy	129	3.5 (3.0 to 4.2)	8645	241.0 (236.0 to 246.2)	1.5 (1.3 to 1.7)
	Latvia	2	1.6 (0.2 to 6.9)	207	174.1 (151.0 to 200.0)	0.9 (0.1 to 3.4)
	Lithuania	8	4.6 (2.0 to 9.4)	344	200.0 (179.3 to 222.6)	2.3 (1.1 to 4.2)
	Netherlands	23	2.9 (1.8 to 4.4)	2333	292.6 (280.9 to 304.8)	1.0 (0.7 to 1.4)
	Portugal	9	1.5 (0.7 to 3.0)	817	138.4 (129.1 to 148.3)	1.1 (0.5 to 2.0)
	Romania	25	2.5 (1.6 to 3.7)	1163	114.0 (107.5 to 120.8)	2.2 (1.5 to 3.0)
	Slovakia	10	4.3 (2.0 to 7.9)	701	291.0 (269.7 to 313.5)	1.5 (0.8 to 2.5)
	Slovenia	7	6.5 (2.6 to 14.0)	369	344.4 (310.1 to 381.7)	1.9 (0.8 to 3.7)
	Sweden	30	5.8 (3.9 to 8.3)	1137	226.7 (213.7 to 240.3)	2.6 (1.8 to 3.5)
	Switzerland	38	9.5 (6.7 to 13.0)	1840	457.6 (436.9 to 479.1)	2.1 (1.5 to 2.7)
	Turkey	12	0.6 (0.3 to 1.1)	3170	140.7 (135.4 to 146.1)	0.4 (0.2 to 0.8)
	United Kingdom	67	2.2 (1.7 to 2.8)	4724	152.6 (148.2 to 157.0)	1.4 (1.1 to 1.8)
	**Overall*** **	**755**	**4.5 (3.4 to 5.6)**	**41125**	**228.3 (194.4 to 262.1)**	**2.0 (1.7 to 2.1)**

TSCI: Traumatic Spinal Cord Injury; CI: Confidence Interval

** We only included those cases where the cause of death has been defined by a specific ICD diagnostic code; cases where the cause was defined as "other and unspecified effects of external causes" or any of the ICD codes of T15-T78 and T80-T88 were excluded*

*** Percent of age-standardized TSCI related mortality rate out of age-standardized all injury related mortality rate*

**** Pooled age-standardized mortality rates estimated using the random effects model*

Figure S1. TSCI-related mortality rates by external cause in 22 European countries in 2012, by sex

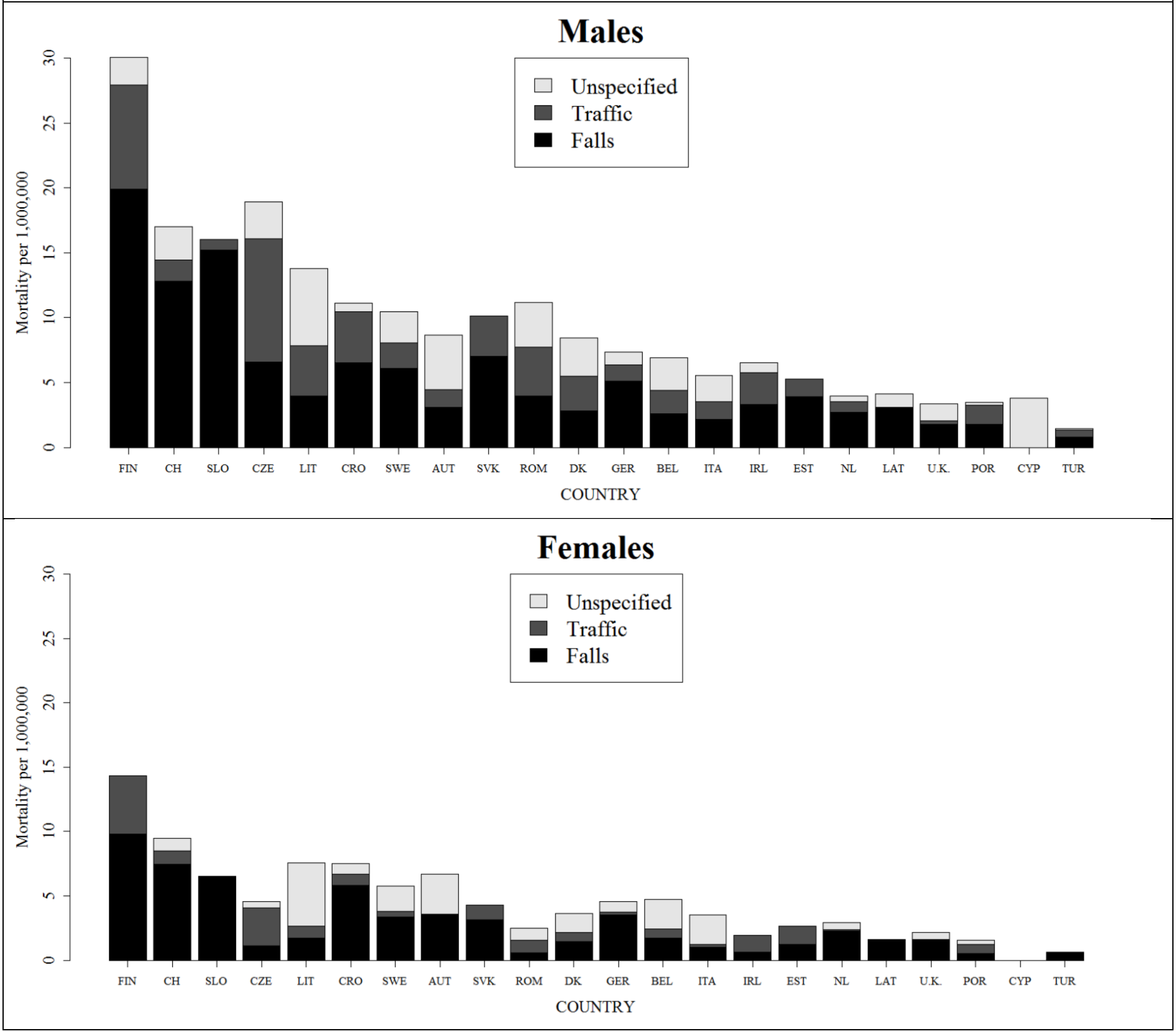

TSCI: Traumatic Spinal Cord Injury

Figure S2. TSCI-related mortality rates by level of injury, in 22 European countries in 2012, by sex

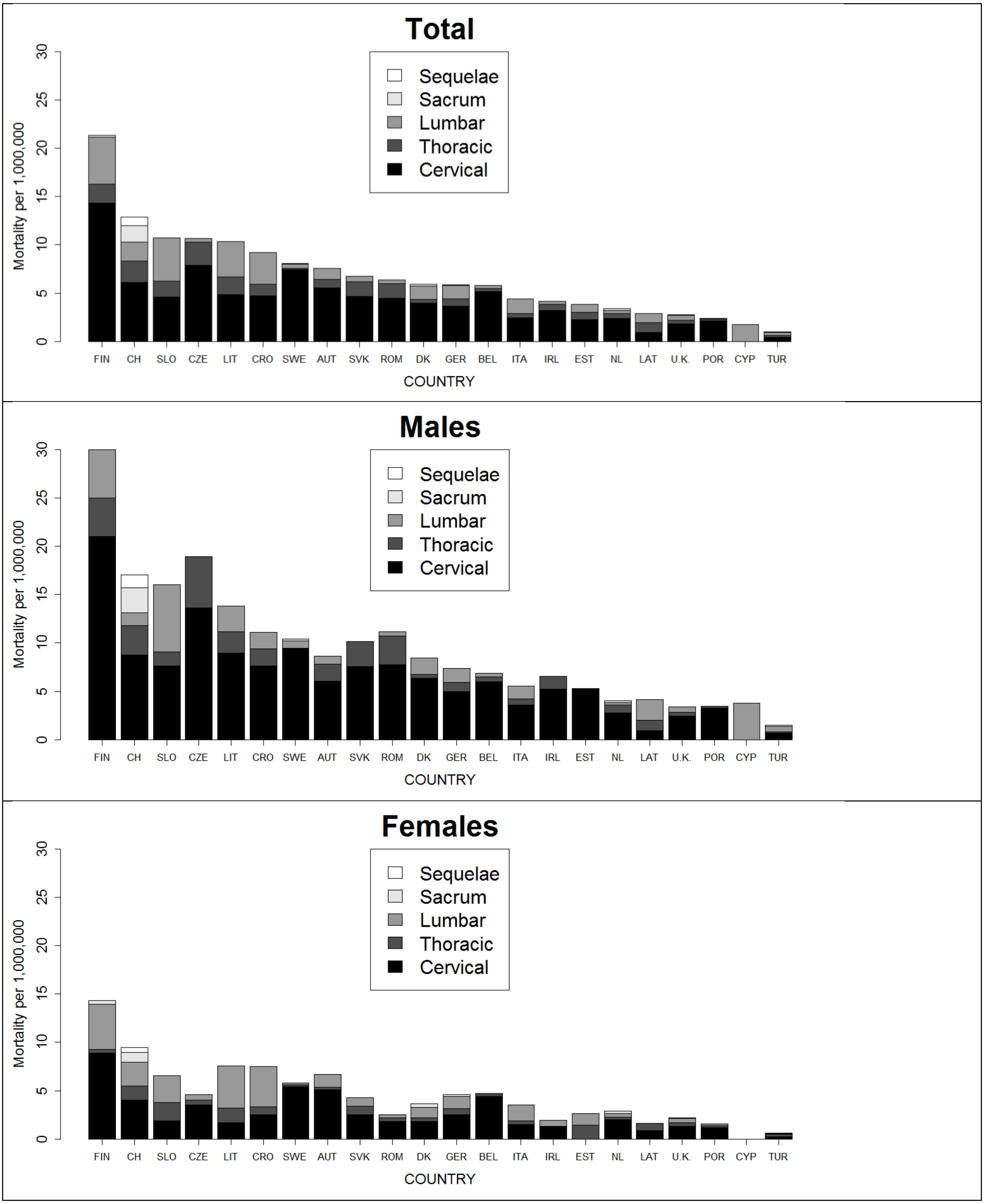

TSCI: Traumatic Spinal Cord Injury
